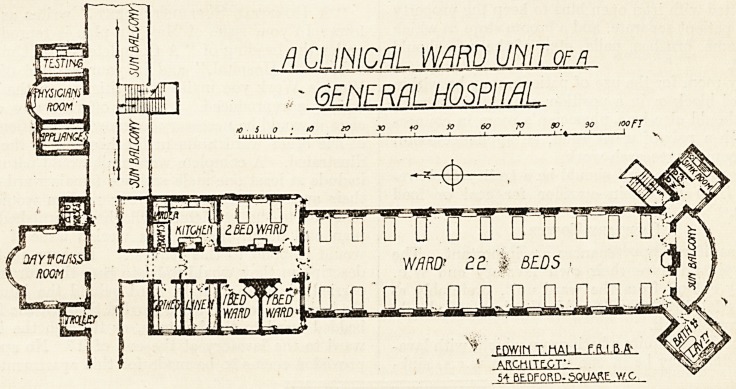# A Complete Ward Unit for a Modern Hospital

**Published:** 1911-05-13

**Authors:** Edwin T. Hall


### Hospital Architecture and Construction.

[Communications on this subject should be marked "Architecture" in the left-hand top corner of the envelope.]

### A COMPLETE WARD UNIT FOR A MODERN HOSPITAL.

By EDWIN T. HALL, F.R.I.B.A., F.R.San.Inst.

The subject of what is most convenient and comprehensive for a clinical ward unit in a general hospital is of importance, as on such elements depend the successful working of the hospital with the minimum of waste in energy and maintenance.

The number of beds to be provided in the unit will vary somewhat in relation to the total number of beds in the hospital. In small hospitals the units are small, but for the present purpose it may be assumed that a large modern general hospital or royal infirmary in an important town is under consideration.

### Number of Beds.

In such large hospitals there is not a consensus of opinion as to the exact number of beds that should be provided in the unit. For example, St. Thomas' Hospital in London has a ward, or rather floor unit, of 28 beds; Derbyshire Royal Infirmary, 26; Liverpool Royal Infirmary, 32; Newcastle Royal Infirmary, 25; Manchester Royal Infirmary, 26.

Abroad there is similar diversity. For examples, the Johns Hopkins Hospital at Baltimore has 28 beds; the Eppendorf at Hamburg (in its largest pavilion), 32; and the Nürnberg, 36.

While, therefore, no dogmatic pronouncement can be made, I have found 25 to 27 beds have been quite satisfactory, and the attached plan shows what I think to be a good plan for general use.

Some hospital wards are made 28 and even 30 feet wide, but 27 feet appears to be liberal, as if 6 feet 6 inches be allowed for a bed, there remains a distance of 14 feet between the beds, ample for every purpose.

If this be so, any greater width involves useless expenditure both in capital and maintenance. It is true that central stoves (generally two) are often placed in the wards, but in my judgment these are undesirable obstructions. One merit claimed for them is that patients can sit around them in comfort. Against this are to be set (1) interference with traffic; (2) they do not warm the air where it is most required namely, near the outer walls and windows; (3) the horizontal flues carried from the stoves to the outer walls are practically rarely swept. In my judgment an open fire at the end of a ward gives the requisite note of cheerfulness, while the effective heating should be done by hot water radiators under windows.

### Dimensions.

The height of the ward is really governed by the æsthetic proportion. For practical purposes sanitarians hold that any air in a room more than 12 feet above a floor is of little or no value, but a ward 27 feet wide and of great length requires a height of more than 12 feet, and this requirement is not only one of taste, but is of therapeutic value, for too low a room has a depressing influence on a patient, and so tends to retard recovery. A minimum of 13 feet 6 inches should be given to such a ward.

The cubic space per patient is one about which much has been written. Personally I am of opinion that in a properly-designed and ventilated ward with windows on both sides this can be deduced automatically from what is otherwise convenient; ample space about and between beds is necessary for staff, etc., and 8 to 10 feet centre to centre along the wall is adequate. This gives, say, 1,400 to 1,800 feet per bed.

In small wards more area is required and 2,000 feet per bed gives a good cubic result.

### Subsidiary Wards.

In addition to the large general ward I think at least two subsidiary attached wards are desirable, one of two beds and one of one bed. These should be in close touch with the large ward, should open out of the ward entrance corridor, and be visible either from the sister's room or the ward kitchen. If, as is sometimes the case, they are placed on the other side of the general hospital corridor, they are not so readily under observation.

The question of a sister's or nurse's sitting-room is one on which authorities differ. Some hold that the nursing staff should be in the ward when on duty and away from it entirely when off duty. It is well to provide the room, as it is always available as a single bed ward if not required for the sister, and may be set apart for sick nurses who are more likely to be well looked after inside a pavilion than in the home. A ward kitchen is essential to the unit, and attached to this should be a larder, preferably entered from the kitchen for convenience and saving of labour. The other accessories are a linen room for the needs of the unit; a patients' clothes room fitted with iron open bins to keep the property of each patient separate, and a broom store in which the brooms, brushes, pails, etc., of the unit should be kept.

A day-room for the use of patients may be within the ward block or in a position convenient thereto. There should also be a physician's room in connection with a pair of ward units, an appliance-room, and a room for research work.

Adjacent to the unit should be a trolley dock, recessed from the main corridor for coal or food trolleys.

### Sanitary Tower.

The sanitary appurtenances are important. The nurses should have their own lavatory and w.c. properly cut off from the ward unit, preferably at the hospital corridor end, and in this lavatory should be a draw-off sink.

The patients require a bath-room which, with lavatory basins, may be in one tower, the w.c.s, sink-room, etc., being in another, both having a proper cut-off. The sink-room should have a macintosh washing sink, a scalding sink, and a bed-pan sink. When there is plenty of room laterally and the ward is not too long these towers are usefully placed at the north end of the large ward, one on each side, and between the western one and the escape staircase at the south end a sun balcony wide enough to take patients may be constructed. This position for the sanitary towers is good if there are several small wards, as it saves a long walk for patients and obviates the necessity of carrying bed-pans all through the long ward. The value of sun balconies is not, I fear, sufficiently appreciated, and it is a good plan to supplement the usual ward balconies by sun balconies off, and sheltered by the main corridor between, the pavilions, as shown on the plan.

In this article I have dealt with a typical ward plan for a general hospital; but of course special hospitals require special treatment. The requirements vary, the nursing is somewhat different, and there are other details which affect the plan.

## Figures and Tables

**Figure f1:**